# Retraction: ASIC1a involves acidic microenvironment-induced activation and autophagy of pancreatic stellate cells

**DOI:** 10.1039/d1ra90106k

**Published:** 2021-04-21

**Authors:** Tao Wang, Qian-qian Wang, Gui-xia Pan, Guo-rong Jia, Xiao Li, Chao Wang, Li-ming Zhang, Chang-jing Zuo

**Affiliations:** Department of Nuclear Medicine, Changhai Hospital, Second Military Medical University Shanghai China changjing.zuo@qq.com; Department of Marine Biotechnology, Faculty of Naval Medicine, Second Military Medical University Shanghai 200433 China lmzhang1969@aliyun.com

## Abstract

Retraction of ‘ASIC1a involves acidic microenvironment-induced activation and autophagy of pancreatic stellate cells’ by Tao Wang *et al.*, *RSC Adv.*, 2018, **8**, 30950–30956, DOI: 10.1039/C8RA03679A.

The Royal Society of Chemistry, with the agreement of the authors, hereby wholly retracts this *RSC Advances* article due to concerns with the reliability of the data in the published article. After publication, the authors contacted the journal to explain that the published western blot images had been edited and arranged because some bands not mentioned in the article had been deleted and the loading order of the samples in the original experimental groups had been adjusted. There is evidence of manipulation affecting the western blot images in Fig. 3A and 4A, and discrepancies in the background of Fig. 6A. The authors confirmed that they do not have the whole raw data for the published western blot images. Although the authors state that they have repeated the experiment and obtained similar results, the concerns over the integrity and validity of the images shown in the published article cannot be dispelled.

Given the significance of the concerns about the validity of the data, and the lack of raw data, the findings presented in this paper are no longer reliable. All of the authors agree with the retraction.

Signed: Tao Wang, Qian-qian Wang, Gui-xia Pan, Guo-rong Jia, Xiao Li, Chao Wang, Li-ming Zhang and Chang-jing Zuo

Date: 7^th^ April 2021

Retraction endorsed by Laura Fisher, Executive Editor, *RSC Advances*

## Supplementary Material

